# Key performance indicators used by dairy consultants during the evaluation of reproductive performance during routine visits

**DOI:** 10.3389/fvets.2023.1165184

**Published:** 2023-06-02

**Authors:** Ramon Armengol, Lorenzo Fraile, Alex Bach

**Affiliations:** ^1^Department of Animal Science, ETSEA, University of Lleida, Lleida, Spain; ^2^Agrotecnio, University of Lleida, Lleida, Spain; ^3^Marlex Recerca i Educació, Barcelona, Spain; ^4^Institució de Recerca i Estudis Avançats (ICREA), Barcelona, Spain

**Keywords:** reproductive performance, routine visit, survey, dairy cattle consultant, key performance indicator

## Abstract

Dairy farms need thorough and efficient reproduction control. Consultants specialized in reproduction use key performance indicators (KPI) to monitor the reproductive performance of farms and must be able to decipher between the approach in a first visit and routine visits. A total of 49 consultants specialized in dairy reproduction from 21 countries responded to an online survey conducted to determine the most suitable parameters during routine visits every 2 to 4 weeks. The survey was comprised of 190 questions, 178 of them rated from 0 (irrelevant) to 10 (maximum importance) points. The questions were divided into five sections: (1) consultant and farm model, (2) general data of the farm, (3) cow reproduction, (4) postpartum and metabolic disease, and (5) heifer reproduction. The median, interquartile range, minimum and maximum values, and 95% confidence interval were determined for each question. Afterward, a multivariate analysis, using between-group linkage via Ward's hierarchical clustering was conducted to generate clusters of consultants according to their response pattern. Finally, a chi-square test was conducted to assess the association between years of experience of the consultant and farm size within the clusters generated in each section of the questionnaire. The majority of the consultants considered 34 parameters to be highly important (rated 8–10) to analyze during routine visits. The consultants used several KPI (in variable quantitative range) to evaluate any of the presented sections and considered that all the five sections are critical to control. They are aware of using KPI that reflect heat detection, fertility, and farming efficiency as well as KPI that can provide information on reproductive efficiency in the near future for cows, such as postpartum and metabolic diseases. However, parameters that are relatively old and ineffective, in terms of reproductive performance control, are still highly regarded by the majority of consultants in a routine-visit scenario. Farm size and years of experience of the consultant did not influence the type or number of parameters chosen as KPI during routine visits. The parameters rated with the highest importance (rate 10) that could be considered for an easy, fast, and universal use in routine visits to assess the reproductive status were: First service CR (%), Overall pregnancy rate (%) for cows, and age at first calving (d) for heifers.

## 1. Introduction

Herd profitability in a dairy farm is highly related to the reproduction efficiency of cows and heifers (1). Thus, achieving pregnancy in cows at the most suitable time is critical for an efficient production system and reduced culling of cows (2–4). Reproduction performance is affected by multiple factors such as nutrition (5), management (6, 7), environment (8, 9), type of the farm (10, 11), technological tools (12, 13) and health (14–18). Farmers are aware of this importance and commonly hire consultants to analyze the reproduction parameters and carry out regular tasks in the farms such as pregnancy diagnosis, prevention of postpartum diseases, therapeutics based on hormones, breeding strategies. and data analysis to maintain all the potential factors affecting reproduction under control (19).

Reproduction consultants must be skilled on most of the former factors and deal with persistent lack of time to tackle the reproduction status of the farm. This time lag is mainly associated with the length of repeated estrus in cows (21 days in average) and the interval of days that a technician needs to accurately diagnose and confirm pregnancy after breeding a cow or a heifer (i.e., 26–30 days) (20, 21). Advising a dairy farm has two distinct stages: (1) the first visit: when the professional relationship with the farmer begins and a first evaluation of the herd's reproductive performance must be conducted (22) and (2) continued and routine visits: when the consultant evaluates the outcomes of the changes implemented, suggests improvements (if needed), sets goals, and identifies and attempts to solve immediate reproductive problems (11).

The main goal of a first-visit scenario should be to obtain an idea reflecting the most recent reproductive situation but also considering the background of the farm. However, the main goal of a routine-visit scenario should be to obtain accurate reproductive data as soon as possible to react to a decrease in reproductive performance in the present or near future.

Today, the reproduction performance of a herd can be calculated by a plenty of parameters that can be obtained on a regular basis from software and databases in farms. They may be used as Key Performance Indicators (KPI). Ironically, the large number of parameters available can lead a consultant to make mistakes on selecting the most suitable KPI to assess the reproductive performance during routine visits. However, consultants' priority should be reducing the negative impact associated with the lag in diagnosing the reproductive problems and implementing corrections.

In this study, we conducted a survey involving several consultants specialized in dairy reproduction from different countries and production systems with the aim to (1) assess the most used parameters to evaluate reproductive performance in a routine visit basis and (2) report critical indicators that, according to the consultants surveyed, may improve the effectiveness of the routine visits to a dairy farm for reproduction consultancy. Thus, the purpose of this study was to (1) describe the KPI that consultants specialized in dairy reproduction use to assess the reproductive status of a conventional dairy farm during routine visits; (2) categorize the different KPIs according to their importance to the consultants; and (3) identify what primary KPI are deemed important by the surveyed consultants and that could be universally used in the routine visit to a conventional dairy farm.

## 2. Materials and methods

### 2.1. Study design

A descriptive and cross-sectional study based on a survey conducted between January 2019 and May 2020 among dairy reproduction consultants located in countries in Europe, America, Asia, Africa, and Oceania was carried out. The survey has been thoroughly described in a previous paper of our research (22). To achieve the most accurate and standardized definitions of the parameters and guarantee the inclusion of the maximum number of KPIs, an online search was performed using the terms dairy cattle, reproduction and efficiency, management, reproductive performance, pathology, advising, and monitoring.

### 2.2. Survey and data collection

Detailed information on the survey and data collection is already published in a research article focused on KPI during a first visit (22). Briefly, the survey was sent by e-mail to 32 national buiatric associations around the world and directly to 22 consultants. The survey did not entail any remuneration, prize, or draw for participants and the responses were anonymous. The survey included a cover letter detailing the objectives of this work and the conditions of participation (placing them on a routine visit scenario—one visit every 2–4 weeks—and assuming that the farm being visited could provide all the parameters included in the survey), along with a link to an online Google Forms questionnaire (for detailed data, see Supplementary Table 1). The power analysis prior to sending the survey indicated that 43 answered questionnaires would be necessary to correctly estimate the mean for each question (CI: 95%, SD = 4, and an accepted absolute error of 1.2) (https://epitools.ausvet.com.au/).

### 2.3. Questionnaire sections

The survey comprised of 190 questions divided into five sections. Each question included a clear definition of each parameter and its calculating equation (if applicable). The complete survey questionnaire is given as Supplementary Table 1.

Briefly, the five sections were (1) consultant and farm model, (2) general data of the farm, (3) cow reproduction, (4) postpartum and metabolic disease, and (5) heifer reproduction.

Sections 2–5 included related parameters that had to be rated by the consultant using an 11-point scale: from 0 (not important at all) to 10 (highly important). To ease the interpretation of the results obtained, the answers were grouped into four categories: Highly relevant (score ≥8), moderately relevant (score between ≥5 and <8), low interest (score between ≥2 and <5), and irrelevant (score < 2).

The term “conception rate” (CR) is used to indicate “conception risk” throughout the manuscript.

### 2.4. Statistical analyses

First, the median, interquartile range, minimum and maximum values (min–max), and confidence interval (CI) of 95% were determined for each question of the questionnaire. Afterward, a multivariate analysis using between-group linkage via Ward's hierarchical clustering was conducted to generate clusters of consultants according to their response pattern, considering the answers from Sections 2–5 of the questionnaire and representing them in a constellation plot. The number of clusters was established by obtaining the best goodness of fit with this multivariate analysis technique. Years of experience and farm size were discretized and analyzed as ordinal variables. Thus, years of experience included the groups: <15, 15–20, 21–25, and >25 years and the size of the farm included the groups: 1–150, 151–300, 301–700, and >700 cows. Finally, a chi-square test was conducted to assess the association between the years of experience of the consultant and farm size within the clusters generated in each section of the questionnaire.

## 3. Results and discussion

A total of 49 consultants from 21 countries answered the questionnaire, averaging only 2.3 consultants per country (range, 1–7). Many countries were not represented, which may pose a significant bias in the data presented. Nevertheless, as discussed later, responders included consultants from several countries at different stages of their careers, and the common characteristics of the farms these consultants advice are quite similar regarding to the production system or geographical area. The results of the survey were split into five sections that are presented below. Two important matters were clearly explained in the cover letter for participants: It was assumed that the respondent was evaluating a dairy farm during a routine visit (every 2 or 4 weeks) and that the consultant could obtain information about all the parameters included in the survey. None of the respondents commented on the impossibility of obtaining any of the parameters included in the suggestions/comments section of the survey.

### 3.1. Section 1: consultant and farm model

The descriptions of the consultants who responded to the survey are detailed in a previous article published by the same authors (22). Briefly, the consultants who responded were from Europe or America, with age ranging from 31 to 70 years and advising closed farms without access to grazing areas for the cows (36; 73.4%), housed in free stalls or cubicles (40; 81.6%), and with a close-up pen (34; 69.4%). The farms mainly comprised Holstein Friesian (HF) cows (35; 71.4%) and used artificial insemination (AI) as the most common breeding technique (47; 95.9%) with year-round calvings (46; 93.8%) and two milkings (33; 67.3%).

Farm size distribution, which referred only to lactating cows, was 1–150 (12; 24.49%), 151–300 (12; 24.49%), 301–700 (13; 26.53%), and >700 (12; 24.49%) and most of the consultants worked with farms milking two times daily (33; 67.3%). Farms with a devoted close-up pen were referenced in 34 of the returned questionnaires (69.4%).

The consultants also answered about the ideal time span of the data they would request to assess reproduction on a routine basis. The results were the same as obtained for a first visit approach (22). Briefly, ~80% of the consultants surveyed would analyze data from the previous year and before. The relevance of these old data for routine visits is not useful to detect and react to reproductive issues in the present or the near future of a dairy farm. However, to consider data from same periods (months or seasons) in the past could be useful to evaluate changes already done and/or be aware of difficult periods of the farm regarding the reproduction performance (i.e., heat stress season). In any case, to considering data older than 3 years should be avoided as the value of that information with regard to the present situation of the farm is low.

### 3.2. Section 2: general data of the farm

The participants could evaluate 43 parameters in this section. The consultants selected six parameters as highly important—pregnant cows (%), culling rate (%), average DIM (n), cows culled for reproductive reason (%), lactating cows daily milk yield (kg) and replacement (%)—30 were moderately important, five were of low interest, and two were considered irrelevant (see Table 1 for detailed data).

**Table 1:** Answers given by consultants for section 2: general data of the farm, where 0 is of minimal or irrelevant importance and 10 represents the maximum importance.

**General data of the farm parameter**	**Median**	**CI 95%**	**Interquartile range**	**Min–max**
Pregnant cows, %	9	5.64–7.86	6.5	0–10
Culling rate, %	8	5.67–7.63	4.0	0–10
Average DIM, d	8	5.94–8.09	4.0	0–10
Cows culled for reproductive reason, %	8	5.98–8.05	4.0	0–10
Lactating cows daily milk yield, kg	8	4.86–6.96	6.0	0–10
Replacement, %	8	5.43–7.66	6.5	0–10
“Do not breed” cows, %	7	5.37–7.27	3.5	0–10
Number of milking cows, *n*	7	5.85–7.41	4.0	0–10
Total number of cows, *n*	7	6.23–7.72	4.5	0–10
Average days dry, d	7	4.67–6.59	4.5	0–10
305 days yield, kg	7	4.56–6.49	5.5	0–10
Peak milk 1st lact cows, kg	7	4.44–6.45	6.0	0–10
Lameness, %	7	4.54–6.60	7.0	0–10
Daily milk yield, kg	7	4.08–6.15	7.5	0–10
Average SCC, SCC/mL	7	4.50–6.60	8.0	0–10
Average lact number of culled cows, *n*	7	4.28–6.44	8.5	0–10
Herd status for BVDV, present/absent	7	4.24–6.52	8.5	0–10
1st lact cows, %	7	4.43–6.67	9.0	0–10
Dry cows, %	7	4.25–6.51	9.0	0–10
Failure to conceive culling rate, %	7	4.44–6.73	9.0	0–10
Number of 1st lact cows, *n*	6	5.23–6.88	3.5	0–10
Peak milk 3rd lact cows, kg	6	4.39–6.38	6.0	0–10
Average DIM culled cows, *n*	6	4.24–6.53	9.0	0–10
Herd status for neosporosis, present/absent	6	3.64–5.74	7.5	0–10
Clinical mastitis, %	6	4.28–6.32	7.5	0–10
Herd status for IBR–IPV, present/absent	6	3.52–5.78	8.0	0–10
Peak milk 3nd lact cows, DIM	6	3.80–5.82	8.0	0–10
Cows culled for lameness reason, %	6	3.90–5.93	8.0	0–10
Cows culled for mastitis reason, %	6	4.06–6.07	8.0	0–10
Peak milk 1st lact cows, DIM	6	4.07–6.12	8.0	0–10
Average number of lact, n	5	4.12–6.07	5.0	0–10
Peak milk >3rd lact cows, kg	5	2.96–5.07	7.0	0–10
Peak milk 2nd lact cows, DIM	5	2.93–5.06	7.5	0–10
Number of dry cows, *n*	5	3.33–5.44	7.5	0–10
Monthly milk yield, kg	5	3.52–5.62	8.0	0–10
Peak milk 2nd lact cows, kg	5	3.59–5.75	8.0	0–10
Cows culled for accident reason, %	4	2.76–4.66	7.0	0–10
All cows daily milk yield, kg	4	3.12–5.19	8.0	0–10
Total number of pregnant cows, *n*	3	2.43–4.46	6.0	0–10
Number of cows culled, *n*	3	2.42–4.43	7.0	0–10
Peak milk >3rd lact cows, DIM	3	2.46–4.51	7.0	0–10
Herd status for FMD, present/absent	0	1.23–3.33	5.0	0–10
Herd status for rucellosis, present/absent	0	2.38–4.80	7.5	0–10

Results are expressed as median, CI 95%, interquartile range and minimum (Min), and maximum (Max) values. Parameters are sorted according to the median obtained, from highest to lowest.

CI, Confidence interval 95%; DIM, Days in milk; lact, lactation; SCC, Somatic cell count; BVDV, Bovine Viral Diarrhea Virus; IBR–IPV, Infectious Bovine Rhinotracheitis—Infectious Pustular Vulvovaginitis; FMD, Foot and Mouth Disease.

The percentage of pregnant cows in the herd was rated with a median of 9, while the other five parameters considered as highly important (rated 8–10) had a median of 8. None of the parameters was rated with a median of 10, reinforcing that the consultants like having an overview on important parameters that involve production and reproduction performance. However, the consultants also seem to be aware that these parameters do not change quickly along time and therefore are not useful to quickly identify or forecast if there is a drop in reproductive performance (none of parameters deserved a median of 10). As an example, % of pregnant cows will provide information about the number of pregnancies in the herd, and this parameter can also be analyzed together with average DIM. However, if the distribution of pregnant cows with regard to the days of pregnancy is not known, then it is impossible to identify any immediate issue affecting fertility.

Parameters involving the turnover rate of the farm (culling and replacement) are also highly important for consultants, as culling for a reproductive reason does. Again, these parameters give an overall picture of the herd, but they change quite slowly, unless a big problem close to a catastrophe occur. Furthermore, at a herd level, culling of a cow is a combination of different reasons. Thus, identifying reproduction based reasons for culling may lead to bias, low accuracy, and a long lag in achieving diagnosis.

The responders were grouped into five clusters for this section (Supplementary Figure 1). Cluster 1 included the highest number of consultants (22; 44.8%), where all the parameters were considered as moderately or highly important with the exception of herd status for the foot and mouth disease (FMD). On the contrary, cluster 5 included 16.3% (*n* = 8) of the consultants that considered that all the parameters in this section were irrelevant. Cluster 2 included 18.4% (*n* = 9) of the consultants that considered 11, 29, 5, and 6 of the parameters were highly important (number of milking cows, lactating cows daily milk yield, % pregnant cows, average DIM, % culling rate, % failure to conceive culling rate, % “do not breed” cows, % cows culled for reproductive reason, % clinical mastitis, % replacement, and average lactation of culled cows), moderately important, and low important or irrelevant, respectively. Clusters 3 and 4 included the lowest number of consultants (6; 12.2% and 4; 8.1%, respectively). In cluster 3, the consultants considered five parameters as highly important (% pregnant cows, % culling rate, % failure to conceive culling rate, % “do not breed” cows, and % cows culled for reproductive reason), 11 as moderately important, 21 as low important, and six as irrelevant. In cluster 4, the consultants considered three parameters as highly important (Number of milking cows, Herd status for BVDV, and Herd status for IBR-IPV), five as moderately important, 17 as low important, and 18 as irrelevant (for detailed data see Supplementary Table 2).

The parameters considered highly important by, at least, 50% of the consultants were: % of pregnant and proportion of cows culled for reproductive reasons cows for 75.5% of the consultants (*n* = 37); average DIM; and % of replacement for 63.2% of the consultants (*n* = 31) (see Table 2 for detailed data).

**Table 2:** Parameters evaluated as highly important (range 8–10) for section 2: general data of the farm used by, at least, 50% of the consultants surveyed after hierarchical clustering analysis.

**General data of the farm**	**Median**	**CI 95%**	**Interquartile range**	**Min–max**	**% of consultants rating as highly important**	**Clusters mainly rating the parameter as highly important**
Pregnant cows, %	9	5.64–7.86	6.5	0–10	75.5 (37)	1, 2, 3
Cows culled for reproductive reason, %	8	5.98–8.05	4.0	0–10	75.5 (37)	1, 2, 3
Average DIM, d	8	5.94–8.09	4.0	0–10	63.2 (31)	1, 2
Replacement, %	8	5.43–7.66	3.5	0–10	63.2 (31)	1, 2

Results are expressed in median, CI 95%, interquartile range, minimum (Min) and maximum (Max) values, and proportion of consultants that rated the parameter as highly important (n in brackets). The clusters obtained after hierarchical clustering analysis where most of these highly important parameters were present are also depicted. Parameters are sorted according to proportion of consultants that rated the parameter as highly important, from highest to lowest.

CI, Confidence interval 95%; DIM, Days in milk.

### 3.3. Section 3: cows' reproduction

This section included 49 parameters—21 were rated as highly important, 22 were considered as moderately important, four were rated as low important, and two were considered irrelevant. Two parameters were rated with the maximum score of 10 (first service CR and overall PR), three parameters obtained a rate of 9 (CR, heat detection rate, and 21 days PR), and the rest were rated at 8 (% abortion >90 days of pregnancy, CR at first service for first lactation cows, % of cows 18–24 days return to service/heat, CR of inseminators, interval service to service, pregnancy loss, calvings per month, days open, services per pregnancy, % of cows conceiving of served, calving to first service interval, herd calving to conception interval, CR multiparous cows, CR first service in multiparous cows, CR first lactation cows, and CR synchronized cows) (see Table 3 for detailed data).

**Table 3:** Answers given by consultants for section 3: cows' reproduction data parameters, where 0 is of minimal or irrelevant importance and 10 represents the maximum importance.

**Cows' reproduction parameter**	**Median**	**CI 95%**	**Interquartile range**	**Min–max**
First service CR, %	10.0	6.53–8.72	3.0	0–10
Overall pregnancy rate, %	10.0	5.81–8.22	7.5	0–10
CR, %	9.0	6.29–8.44	3.5	0–10
Heat detection rate, %	9.0	6.06–8.29	4.0	0–10
21d. Pregnancy rate, %	9.0	5.29–7.80	10.0	0–10
Abortion >90 days, %	8.0	5.50–7.51	3.5	0–10
CR first service in 1st lact cows, %	8.0	5.81–8.01	5.0	0–10
18–24 d. return to service/heat, %	8.0	4.97–7.10	5.5	0–10
CR of inseminators, %	8.0	5.03–7.12	6.0	0–10
Interval service to service, d	8.0	5.33–7.48	6.0	0–10
Pregnancy loss, %	8.0	5.31–7.46	6.5	0–10
Calvings per month, *n*	8.0	4.96–7.07	7.0	0–10
Days open, *n*	8.0	5.29–7.52	7.5	0–10
Services per pregnancy, *n*	8.0	4.85–7.14	8.5	0–10
Conceiving of served, %	8.0	4.76–7.11	9.0	0–10
Calving to first service interval, d	8.0	4.98–7.22	9.0	0–10
Herd calving to conception interval, d	8.0	4.49–6.93	10.0	0–10
CR multiparous cows, %	8.0	4.94–7.25	10.0	0–10
CR first service in multiparous cows, %	8.0	5.08–7.44	10.0	0–10
CR 1st lact cows, %	8.0	5.17–7.51	10.0	0–10
CR synchronized cows, %	8.0	5.22–7.59	10.0	0–10
Voluntary waiting period, d	7.0	5.52–7.50	4.0	1–10
Pregnancy loss (1–90 days), %	7.0	4.68–6.66	5.5	0–10
CR of the sire, %	7.0	4.49–6.61	7.0	0–10
25–35 d. return to service/heat, %	7.0	4.13–6.27	7.5	0–10
1st lact cows calved, %	7.0	4.21–6.23	8.0	0–10
Interval heat to heat, d	7.0	4.28–6.49	9.0	0–10
Cows not pregnant >200 DIM, %	7.0	4.36–6.69	9.0	0–10
CR first Service in 2nd lact cows, %	7.0	4.27–6.62	10.0	0–10
Days at pregnancy diagnosis, n	6.0	4.35–6.34	6.5	0–10
Submission rate first 3 weeks, %	6.0	3.76–6.03	8.0	0–10
Anovulatory cows, %	6.0	4.03–6.21	8.5	0–10
Calving interval, d	6.0	4.06–6.18	8.5	0–10
Cows not pregnant >150 DIM, %	6.0	4.00–6.27	8.5	0–10
Ovarian cysts, %	5.0	3.16–5.32	7.5	0–10
Cows served < 90 DIM, %	5.0	2.93–5.18	8.0	0–10
CR 3rd lact cows, %	5.0	3.19–5.41	8.0	0–10
CR first service in >3rd lact cows, %	5.0	3.41–5.60	8.0	0–10
CR 2^nd^ lact cows, %	5.0	3.51–5.82	8.0	0–10
>49 d. return to service/heat, %	5.0	3.68–5.86	8.0	0–10
CR first Service in 3rd lact cows, %	5.0	3.71–6.03	8.0	0–10
2–17 d. return to service/heat, %	5.0	3.79–5.80	8.0	0–10
36–48 d. return to service/heat, %	5.0	3.88–5.99	8.0	0–10
Early pregnancy loss (1–42 days), %	4.0	2.94–5.17	8.0	0–10
Submission rate, %	4.0	2.77–4.98	8.0	0–10
CR >3rd lact cows, %	4.0	2.80–4.95	8.0	0–10
Non-return rate, %	2.0	2.18–4.18	7.0	0–10
Days to culling, *n*	1.0	1.77–3.53	5.0	0–8
100–Day In–calf rate, %	0.0	2.13–4.27	7.0	0–10

Results are expressed as median, CI 95%, interquartile range and minimum (Min) and maximum (Max) values. Parameters are sorted according to the median obtained, from highest to lowest.

CI, Confidence interval 95%; CR, Conception rate; DIM, Days in milk; lact, lactation.

Essentially, the parameters evaluated as highly important should be sufficient to accurately evaluate the reproductive performance and forecast its trend (for the next 9 months). In a routine-visit scenario, consultants should fulfill two priority objectives: confirm, as soon as possible, the guarantee of enough and homogeneous number of pregnancies at the desired time and forecast possible negative impacts on reproduction in the future.

In evaluating the achievement of pregnancies, there will always be a lag (26–39 days post-breeding/insemination) in confirming pregnancies through the pregnancy check. Consultants should be aware of this lag and should use the most reliable KPIs that directly or indirectly provide the most accurate view to confirm pregnancies. It means that those KPI should be used to analyze data related to the absence of repeated heat after insemination and when these repeated heats occur. Therefore, a continuous (weekly) analysis of heat detection and reinsemination parameters is appropriate and should be a priority to forecast whether pregnancies will be achieved and confirmed. The confirmation of conception and evaluation of its efficiency (getting the cows pregnant as soon as possible after the VWP) is also important and hence it is logical that consultants give high value to CRs (including overall CR, in different group of cows and different factors that can immediately affect CR such as the technicians involved in insemination or synchronization protocols) even if there is a lag. Traditionally, CRs have been controlled monthly. However, with the data immediately available through the software and the increasing size of herds, it is highly recommended that CRs be analyzed weekly (ideally after the pregnancy check).

Another factor that has a direct impact on conception is the different sires used for insemination (23). Surprisingly, the CR of the sire was rated 7 (moderately important). This could be due to different reasons; for example, in small farms, it is difficult to evaluate the fertility of different sires at the same time period, since only one or very few sires are used during the same period and the number of inseminations is too low to draw statistical conclusions. In large herds, the difficulty of gathering and organizing data pertaining to the sires' CR may be a potential reason for the low importance assigned in the survey. That is, in large farms, technicians do so many inseminations from so many different sires that by the time they can observe the CR of a particular sire, those semen doses have been used up and other sires are being used. However, we believe that the CR of the sire should be considered as highly important and could indirectly be analyzed though the reinsemination KPI of cows bred with a particular sire. It is also logical and interesting that consultants analyze the pace of achieving pregnancies in the herd. These parameters consider and combine information about heat detection and conception.

Furthermore, analyzing factors that can have a direct impact on breeding efficacy, such as sire or technician (23, 24), should include a multifactorial analysis considering all the variables (i.e., type of cow (first lactation vs. multiparous), the number of insemination, or whether under hormonal treatment). When analyzing the technician CR, avoiding this multivariable analysis at a farm level, might be not that biased when all the technicians involved are working in a normal distribution of days and on the same group of cows.

What is not useful at all, during a routine visit scenario, is to consider as highly important KPIs that need a lot of time to be obtained, that only include a certain group of cows, or reflect problems from the past, not useful to evaluate the present or near future performance (i.e., open days or calvings per month). Other KPI that indirectly reflect CR or heat detection (i.e., services per pregnancy or interval service to service, respectively) could also be considered as highly important. The only reason these parameters could be included in the routine of KPI analysis would be the farmers' request to include them since they have been used for a long time and they would be very useful to them. Even in this situation, the consultant should warn the farmer about the limitations of these KPI for the routine and continuous control of reproductive performance. Considering calving to first service as highly important would be only logical and useful in farms where the VWP is set and synchronizing protocols are not used for first service. In these farms, consultants should consider that calving to first service is very similar to VWP (average in days) and there are no cows bred before the VWP (distribution). Assuming a normal distribution of heat, calving to first service should approximately be between VWP + 13–13 days. In farms under synchronized first service, the interval calving to first service is always fixed and with the same value than VWP. Thus, the only reason to monitor this KPI in first service synchronized farms would be to control that the protocol is well-implemented.

Finally, consultants rated highly important parameters include % abortion >90 days of pregnancy and pregnancy loss. Although there is an obvious lag (need of two or three pregnancy diagnosis and confirmations), it is always interesting to monitor pregnancy loss or abortions and should be carried out as a routine. Since it is the only way to evaluate the possibility of abortion/pregnancy loss outbreaks and prevent endemic (infectious or not) issues in cows to prevent pregnant or technician mistakes during the pregnancy diagnosis. As an example, a not well-trained veterinarian could be diagnosing cows pregnancy with ultrasonography at 28–35 days post AI that are really not pregnant (low knowledge) and/or making them lose pregnancy with appropriate/traumatic manipulation (low technical skill). This mistake would increase the number of heat-showing “pregnant” cows and/or increase the number of “aborted” cows 90 days after the confirmation of pregnancy by AI (25). In large farms or veterinarian groups, those with more than 1 technician doing ultrasonography, it is quite easy to identify non-skilled technicians when pregnancy check results are compared as a routine. But in case there is only one technician, this “iatrogenic” problem can become endemic in the farm; unless a good and continuous training is place.

After the hierarchical analysis of the answers for Section 3 (see Supplementary Figure 2), the consultants were grouped into five clusters. Clusters 1, 5, and 2 represented 79.6% of the respondents (*n* = 39). The major cluster (1) accounted 46.9% of the consultants surveyed (*n* = 23) and most of the parameters were rated as highly (26) or moderately (15) important with the exception of days at culling that was considered as low important. Cluster 2 included 14.3% of the consultants (*n* = 7) and rated 24, 13, 11, and 1 of the parameters as highly, moderately, and low important and irrelevant, respectively. Cluster 3 was the most reduced one, representing 8.1% of the respondents (*n* = 4), where 19 parameters were considered as highly important, 14 as moderately important, 13 as low important, and three as irrelevant. In cluster 4, representing 12.2% of the consultants (*n* = 6), the majority of the parameters were rated as moderately (25) or low (17) important, seven parameters were rated as highly important and none was considered irrelevant. Cluster 5 represented 18.4% of the surveyed consultants (*n* = 9) who surprisingly rated 12 parameters as low important and 37 as irrelevant. Overall, the top-rated parameter was CR at first service (for detailed data, see Supplementary Table 3). It is logical, since it is the main goal of farmers and consultants to achieve pregnancies as soon as possible and at a lower breeding cost. Other most rated parameters were CRs of first lactation cows and multiparous rather than CRs split by number of lactation (first, second, third, or more than third). This could seem logical, assuming that first lactation cows have a different physiology (and therefore, reproductive performance and management) compared to multiparous cows, since they are still growing and developing to become a mature cow (27–29). However, we believe that routine consultancy should include a deeper analysis of all groups of cows with regard to the number of lactations. Analyzing CRs for each lactation could be of more benefit because the economical factor could be more controlled as most of the second lactation cows are not paid back yet with regard to the investment required during the heifer rearing process (30). Parameters that reflect heat detection, late abortion (>90 days of pregnancy), and cows pregnant at the most profitable period of time (< 150 DIM) were also the most rated.

Taking into account the highest averages obtained from the evaluated parameters, rated as highly important by, at least, 50% of the respondents, that could be considered the most important KPIs for this section are as follows: first service CR was considered highly important by the 81.6% of the consultants (*n* = 40); the overall pregnancy rate, CR, heat detection rate, and days open by 73.5% (*n* = 36); abortion >90 days of pregnancy, CR at first service in first lactation cows, 18–24 days return to service/heat, interval service to service, calving to first service interval, CR in multiparous cows by 69.4% of the consultants (*n* = 34); percentage of cows conceiving of served by 67.3% (*n* = 33); finally, 21 days pregnancy rate, pregnancy loss, calvings per month, services per pregnancy, CR in multiparous cows, CR of inseminators, and CR of synchronized cows by 61.2% of the consultants (*n* = 30) (see Table 4 for detailed data).

**Table 4:** Parameters evaluated as highly important (range 8–10) for section 3: cows' reproduction used by, at least, 50% of the consultants surveyed after hierarchical clustering analysis.

**Cows' reproduction parameter**	**Median**	**CI 95%**	**Interquartile range**	**Min–max**	**% of consultants that rated the parameter as highly important**	**Clusters mainly rating the parameter as highly important**
First service CR, %	10.0	6.53–8.72	3.0	0–10	81.6 (40)	1, 2, 3, 4
Overall pregnancy rate, %	10.0	5.81–8.22	7.5	0–10	73.5 (36)	1, 2, 4
CR, %	9.0	6.29–8.44	3.5	0–10	73.5 (36)	1, 2, 4
Heat detection rate, %	9.0	6.06–8.29	4.0	0–10	73.5 (36)	1, 2, 4
Days open, n	8.0	5.29–7.52	7.5	0–10	73.5 (36)	1, 2, 4
Abortion >90 days, %	8.0	5.50–7.51	3.5	0–10	69.4 (34)	1, 2, 3
CR first service in 1st lact cows, %	8.0	5.81–8.01	5.0	0–10	69.4 (34)	1, 2, 3
18–24 d. return to service/heat, %	8.0	4.97–7.10	5.5	0–10	69.4 (34)	1, 2, 3
Interval service to service, d	8.0	5.33–7.48	6.0	0–10	69.4 (34)	1, 2, 3
Calving to first service interval, d	8.0	4.98–7.22	9.0	0–10	69.4 (34)	1, 2, 3
CR first service in multiparous cows, %	8.0	5.08–7.44	10.0	0–10	69.4 (34)	1, 2, 3
Conceiving of served, %	8.0	5.57–7.56	4.5	0–10	67.3 (33)	1, 3, 4
21d. Pregnancy rate, %	9.0	5.29–7.80	10.0	0–10	61.2 (30)	1, 2
Pregnancy loss, %	8.0	5.31–7.46	6.5	0–10	61.2 (30)	1, 2
Calvings per month, *n*	8.0	4.96–7.07	7.0	0–10	61.2 (30)	1, 2
Services per pregnancy, *n*	8.0	4.85–7.14	8.5	0–10	61.2 (30)	1, 2
CR multiparous cows, %	8.0	4.94–7.25	10.0	0–10	61.2 (30)	1, 2
CR of inseminators, %	8.0	5.03–7.12	6.0	0–10	61.2 (30)	1, 2
CR synchronized cows, %	8.0	5.75–7.79	5.5	0–10	61.2 (30)	1, 2

Results are expressed as median, CI 95%, interquartile range, minimum (Min) and maximum (Max) values, and proportion of consultants that rated the parameter as highly important (number of observations within brackets). The clusters obtained after hierarchical clustering analysis where most of these highly important parameters were present are also depicted. Parameters are sorted according to the proportion of consultants that rated the parameter as highly important, from highest to lowest.

CI, Confidence interval, 95%; CR, Conception rate; lact, lactation.

Cows do get pregnant when the combination of a good probability to be inseminated (heat detection, hormonal synchronization, or both) and CR is high (3). Therefore, parameters that monitor CRs and heat detection as fast as possible are deemed essential by most of the consultants. Respondents seem also to be aware that another critical point to continuously analyze is to get cows pregnant efficiently and at a regular pace to maximize the reproductive performance of the farms, considering as highly important all CRs referred to first service, pregnancy rates, and CRs that control external factors of cows such as the inseminator and the hormonal protocols. Finally, consultants want to continually update pregnancy loss events. Especially, regarding late pregnancy since it is economically the least desirable and the one that most compromises the survival of the cow in the herd (31, 32). Furthermore, if pregnancy loss is high and continued over time, farm replacement can be seriously compromised. In other words, the consultant must always be aware of what (infectious or not) may be causing high pregnancy losses and try to solve it as quickly as possible as future direct and indirect economic losses can be very high over a long period of time.

To sum up, more than 50% of the consultants surveyed considered essential a total of 19 parameters and rated them as highly important. However, we consider that there is an overuse of parameters that are not very useful, not reliable, and that may lead to important bias, such as days open or parameters that are indirectly giving information already known with the CRs or pregnancy rate, such as number of services per pregnancy.

### 3.4. Section 4: postpartum and metabolic diseases

Of the 36 parameters, six included in this section were considered as highly important, 13 were considered as moderately important, 12 were rated as low important, and seven were considered irrelevant. Consultants considered the following factors as highly important to monitor in a routine basis: metritis, hypocalcaemia, clinical and subclinical ketosis, and retained placenta (see Table 5 for detailed data). It is very well-known that the four diseases can have a negative impact reproductive performance (15, 26, 33, 34) affecting the most profitable time to get a cow pregnant (<200 DIM) (35). We consider the difference between these results and those obtained from the same consultants but where they were asked to evaluate the parameters in a scenario of a first visit to a farm in which the reproductive status was unknown as remarkable and of special interest (22). In the first visit scenario, the respondents did not consider any of the parameters in this section to be highly important. This difference is logical as postpartum and metabolic diseases have a future impact on reproduction but provide little information about the current reproduction situation. We believe it is important to highlight this difference as it shows that the majority of surveyed consultants are able to differentiate the parameters in each of the scenarios (first visit *vs*. continued and routine visit).

**Table 5:** Answers given by consultants for section 4: postpartum and metabolic diseases parameters, where 0 is of minimal or irrelevant importance and 10 represents the maximum importance.

**Postpartum and metabolic disease parameter**	**Median**	**CI 95%**	**Interquartile range**	**Min–max**
Metritis, %	9.0	5.50–7.67	5.0	0–10
Hypocalcaemia, %	8.0	5.03–7.24	6.5	0–10
Clinical and subclinical ketosis, %	8.0	5.09–7.31	7.0	0–10
Retained placenta, %	8.0	5.24–7.48	6.5	0–10
Clinical ketosis, %	7.0	4.09–6.39	9.0	0–10
Retained placenta multiparous, %	6.0	3.62–5.88	8.0	0–10
Clinical and subclinical ketosis multiparous, %	6.0	3.63–5.99	9.0	0–10
Hypocalcaemia multiparous, %	6.0	3.79–6.16	9.0	0–10
Abomasal pathology, %	6.0	4.12–6.11	8.0	0–10
Stillbirth, %	6.0	4.12–6.16	7.0	0–10
Stillbirth 1st lact, %	5.0	2.93–5.10	8.0	0–10
Metritis multiparous, %	5.0	3.37–5.60	8.0	0–10
Clinical and subclinical ketosis 1st lact, %	5.0	3.35–5.54	8.0	0–10
Retained placenta 1st lact, %	5.0	3.30–5.59	8.0	0–10
Twins, %	5.0	3.51–5.46	7.5	0–10
Dystocia, %	5.0	3.55–5.70	8.0	0–10
Metritis 1st lact, %	5.0	3.69–5.97	8.5	0–10
Hypocalcaemia 1st lact, %	4.0	2.66–4.76	7.0	0–10
Stillbirth multiparous, %	4.0	2.71–4.71	7.0	0–10
Twins multiparous, %	4.0	2.72–4.66	7.0	0–10
Clinical ketosis 1st lact, %	4.0	2.77–4.86	7.5	0–10
Clinical ketosis multiparous, %	4.0	2.90–5.26	8.0	0–10
Dystocia 1st lact, %	4.0	2.94–5.09	7.5	0–10
Abomasal pathology 1st lact, %	3.0	2.37–4.40	7.0	0–10
Dystocia multiparous, %	3.0	2.49–4.48	7.0	0–10
Abomasal pathology multiparous, %	3.0	2.54–4.56	7.0	0–9
Pyometra, %	3.0	2.64–4.74	7.5	0–10
Twins 1st lact, %	2.0	1.89–3.65	5.0	0–10
Incorrect uterine involution after 30DIM, %	2.0	2.63–4.87	8.0	0–10
Pyometra multiparous, %	1.0	1.67–3.61	6.0	0–8
Pyometra 1st lact, %	1.0	1.80–3.66	6.0	0–10
Perineal injury, %	1.0	1.95–3.84	6.0	0–9
Incorrect uterine involution after 30DIM 1st lact, %	0.0	1.61–3.61	6.0	0–10
Perineal injury multiparous, %	0.0	1.15–2.63	4.0	0–8
Incorrect uterine involution after 30 DIM multiparous, %	0.0	1.67–3.70	5.5	0–9
Perineal injury 1st lact, %	0.0	1.70–3.56	5.5	0–10

Results are expressed as median, CI 95%, interquartile range and minimum (Min) and maximum (Max) values. Parameters are sorted according to the median obtained, from highest to lowest.

CI, Confidence interval 95%; DIM, Days in milk; lact, lactation.

Consultants were grouped into five clusters in this section (see Supplementary Figure 3). The proportion of each cluster was 30.6% (*n* = 15), 4.1% (*n* = 2), 30.6% (*n* = 15), 16.3% (*n* = 8) and 18.4% (*n* = 9) for clusters 1, 2, 3, 4 and 5, respectively. It is interesting to highlight that cluster 1 considered all the parameters as highly or moderately important, whereas cluster 5 considered all the parameters as irrelevant (see Supplementary Table 4). This remarkable difference in priorities regarding postpartum and metabolic diseases can be due to several reasons. One reason could be that consultants in cluster 5 of this section focus on strict reproduction KPI from other sections and, unless they have the suspicion that postpartum and metabolic diseases are causing a negative reproductive performance issue, they do not check these parameters as KPIs on a routine basis. They would then analyze this information on a timely basis. Another possibility could be that consultants in cluster 5 just ignore the importance of the impact that postpartum and metabolic diseases might have on the reproduction performance in the near future. But we really consider that this is not the case because this impact is very well-known and demonstrated many years ago and is agreed by a considerable number of researchers (33).

After hierarchical clustering, a total of 25 consultants (51.0%) belonging to clusters 1, 2 and 4 considered it highly important to analyze routinely and continuously four parameters: % of metritis, % hypocalcaemia, % clinical and subclinical ketosis, and % retained placenta (see Table 6 for detailed data). Regarding these results, the consultants surveyed do not consider it necessary to analyze these data according to the different number of lactations or between primiparous or multiparous cows. It would seem logical for consultants to focus their attention on the incidence of diseases on cows in general, without differentiating between first lactation cows or multiparous cows. The consultants probably check these parameters and only analyze the different groups in detail, if the overall incidence is too high according to their criteria. This strategy has advantages and disadvantages: the main advantage is that the consultant saves time by obtaining the data and analyzing them (i.e., analysis of % metritis of all cows) as he does not have to duplicate information (i.e., analysis of % metritis in first lactation cows and % of metritis in multiparous). The disadvantage is that some of these postpartum and metabolic diseases require quick and effective solutions to reduce the impact they can have on reproduction, the worsening of their health, or even death (i.e., acute puerperal metritis consequence of an untreated clinical metritis or retained placenta or left displaced abomasum consequence of a ketosis). Furthermore, the control and prevention of such diseases involves a lot of areas in a farm (i.e., nutrition, management, and treatments) (36, 37). Thus, although analyzing these four diseases in the two different groups of cows (primiparous and multiparous) requires a greater investment of time, it is recommended and is beneficial to solve postpartum and metabolic diseases problems as soon as possible in a farm.

**Table 6:** Parameters evaluated as highly important (range 8–10) for section 4: postpartum and metabolic diseases parameter used by, at least, 50% of the consultants surveyed after hierarchical clustering analysis.

**Postpartum and metabolic diseases parameter**	**Median**	**CI 95%**	**Interquartile range**	**Min–max**	**% of consultants that rated the parameter as highly important**	**Clusters mainly rating the parameter as highly important**
Metritis, %	9.0	5.50–7.67	5.0	0–10	51.0 (25)	1, 2, 4
Hypocalcaemia, %	8.0	5.03–7.24	6.5	0–10	51.0 (25)	1, 2, 4
Clinical and subclinical ketosis, %	8.0	5.09–7.31	7.0	0–10	51.0 (25)	1, 2, 4
Retained placenta, %	8.0	5.24–7.48	6.5	0–10	51.0 (25)	1, 2, 4

Results are expressed as median, CI 95%, interquartile range, minimum (Min) and maximum (Max) values, and proportion of consultants that rated the parameter as highly important (number of observations within brackets). The clusters obtained after hierarchical clustering analysis where most of these highly important parameters were present are also depicted. Parameters are sorted according to the proportion of consultants that rated the parameter as highly important, from highest to lowest.

CI, Confidence interval 95%.

### 3.5. Section 5: heifers' reproduction

This section had 50 parameters to be evaluated. We believe it is important to emphasize that some of these parameters were redundant, as they called for the evaluation of the same parameter but with different numerical values that brought to obtain a lot of irrelevant parameters. As an example, consultants had to evaluate the proportion of heifer calving before 21, 22, 23, or 24 months old.

Seven parameters in this section were considered as highly important (age at first calving, CR at first service, CR, CR of inseminators, heifers culled for reproductive reasons, age at first service, and heat detection). Nineteen parameters were rated as moderately important and 24 as irrelevant. The most consistent and robust KPI of this section was age at first calving, rated with a 10.0, and a low interquartile range (see Table 7).

**Table 7:** Answers given by consultants for section 5: heifers' reproduction parameters, where 0 is of minimal or irrelevant importance and 10 represents the maximum importance.

**Heifers' reproduction parameters**	**Median**	**CI 95%**	**Interquartile range**	**Min–max**
Age at 1st calving, d	10.0	6.46–8.63	3.0	0–10
First service CR, %	9.0	5.56–7.90	8.0	0–10
CR, %	9.0	6.62–8.71	3.0	0–10
CR of Inseminators, %	8.0	4.84–7.11	9.0	0–10
Heifers culled for reproductive reason, %	8.0	5.23–7.54	8.5	0–10
Age at first service, d	8.0	5.39–7.66	7.5	0–10
Heat detection rate, %	8.0	5.56–7.77	5.5	0–10
Heifers calving < 23 months old, %	7.0	3.34–5.79	8.0	0–10
Heifers >14 months old not serviced, %	7.0	3.58–6.04	8.5	0–10
Heifers/cows, %	7.0	3.84–6.15	8.0	0–10
% “do not breed” heifers, %	7.0	3.88–6.15	8.0	0–10
Pregnancy loss (1–90 days), %	7.0	3.96–6.31	7.0	0–10
Anovulatory heifers, %	7.0	4.04–6.32	8.0	0–10
Abortion >90 days, %	7.0	4.06–6.50	9.0	0–10
CR of the sire, %	7.0	4.06–6.46	9.0	0–10
21 d pregnancy rate, %	7.0	4.29–6.81	10.0	0–10
Interval service to service, d	7.0	4.43–6.79	9.0	0–10
Culling rate heifers, %	7.0	4.57–6.68	6.5	0–10
Pregnancy loss, %	7.0	4.61–6.89	9.0	0–10
Services per pregnant heifer, *n*	7.0	4.80–7.19	9.5	0–10
Interval heat to heat, d	6.0	3.92–6.20	8.0	0–10
Heifers calving < 24 months old, %	6.0	3.56–6.07	9.0	0–10
Heifers pregnant, %	6.0	3.75–6.16	9.0	0–10
CR synchronized heifers, %	6.0	3.84–6.19	8.0	0–10
Number of heifers, *n*	5.0	2.83–5.08	8.0	0–10
Days at pregnancy diagnosis, d	5.0	3.32–5.28	7.5	0–10
Heifers < 12 months old, %	2.0	2.35–4.50	7.5	0–10
Heifers >12 months old, %	1.0	2.25–4.40	7.0	0–10
Heifers >11 months old not serviced, %	0.0	0.43–1.48	1.5	0–8
Heifers >11 months old, %	0.0	0.62–2.07	1.5	0–10
Open heifers > 12 months, %	0.0	0.63–2.18	1.5	0–9
Heifers >12 months old not serviced, %	0.0	0.67–2.01	2.0	0–8
Heifers < 11 months old, %	0.0	0.73–2.32	2.5	0–10
Open heifers > 13 months, %	0.0	0.90–2.68	3.5	0–10
Heifers >13 months old, %	0.0	0.90–2.60	4.0	0–10
Heifers < 13 months old, %	0.0	0.96–2.74	4.5	0–10
Heifers >13 months old not serviced, %	0.0	1.06–2.81	4.0	0–9
Heifers < 2 standard deviations from 400 kg at 400 d, %	0.0	1.06–3.01	5.0	0–10
Heifer efficiency, %	0.0	1.27–3.05	5.0	0–10
Heifers calving < 21 months old, %	0.0	1.43–3.50	6.0	0–10
Open heifers > 14 months, %	0.0	1.53–3.72	6.0	0–10
Ovarian cysts, %	0.0	1.75–3.75	5.0	0–10
Heifers < 580 kg at calving, %	0.0	1.83–4.16	8.0	0–10
Early pregnancy loss (1–42 days), %	0.0	1.85–4.10	7.0	0–10
Open heifers > 15 months, %	0.0	1.87–4.24	8.0	0–10
Heifers calving < 22 months old, %	0.0	1.91–4.04	6.0	0–10
Heifers >14 months old, %	0.0	1.99–4.12	7.0	0–10
Open heifers > 17 months, %	0.0	2.16–4.61	8.0	0–10
Heifers < 14 months old, %	0.0	2.25–4.39	7.0	0–10
Open heifers > 16 months, %	0.0	2.46–4.96	9.0	0–10

Results are expressed as median, CI 95%, interquartile range and minimum (Min) and maximum (Max) values. Parameters are sorted according to the median obtained, from highest to lowest.

CI, Confidence interval 95%; CR, Conception rate.

Consultants focused on one of the gold standard KPI to evaluate not only reproductive performance but also heifer rearing process (age at first calving). This is logical, since this parameter also reflects well the efficiency of the heifers' reproductive strategy of a farm. However, age at first calving provides quite old information regarding reproduction (9 months ago), and consultants specialized on reproduction should be expected to prioritize other parameters such as age at pregnancy, much more actual and reliable than age at first calving. Unfortunately, due a technical issue, the item “age at pregnancy” was not shown in the survey and could not be evaluated by the consultants. All of the consultants claimed for this parameter in the final space of the section, where they could write any comment/question. In any case, when consultants are analyzing such parameters that reflect averages, they should always take into account the degree of the spread of data, including the standard deviation (38).

Answered surveys were grouped into five clusters (see Supplementary Figure 4). Cluster 1 represented 28.6% of the consultants (*n* = 14) considering 25 parameters as highly important. Clusters 2 and 5 included 20.4% of the consultants each (*n* = 10) and rated 27 and 0 parameters as highly important, respectively. Actually, cluster 5 considered all parameters as irrelevant with the exception of age at first calving and CR. Cluster 3 represented 18.4% of the consultants (*n* = 9) and rated 11 parameters as highly important. Finally, cluster 4 represented the lowest proportion of consultants surveyed (5; 10.2%), where 22 parameters were considered as highly important (see Supplementary Table 5). Clusters 1, 2, 3, and 4 agreed that CR and age at first calving are highly important, while in cluster 5 they were the only two parameters that were not considered as irrelevant and rated them as low important, thus confirming that consultants not only prioritize getting heifers pregnant but also parameters related to the efficiency of the whole rearing process and achieving first calving as soon as possible.

In this section, seven parameters were considered highly important for, at least, 50% of the consultants: age at first calving, CR, heat detection, and CR of the inseminators for 77.6% of the consultants (*n* = 38); first service CR, age at first service, and heifers culled for reproductive reasons for 59.2% of the consultants (*n* =2 9) (see Table 8).

**Table 8:** Parameters evaluated as highly important (range 8–10) on section 5: heifer reproduction used by, at least, 50% of the consultants surveyed after hierarchical clustering analysis.

**Heifers' reproduction parameter**	**Median**	**CI 95%**	**Interquartile range**	**Min–max**	**% of consultants that rated the parameter as highly important**	**Clusters mainly rating the parameter as highly important**
Age at 1st calving, d	10.0	6.46–8.63	3.0	0–10	77.6 (38)	1, 2, 3, 4
CR, %	9.0	6.62–8.71	3.0	0–10	77.6 (38)	1, 2, 3, 4
Heat detection rate, %	8.0	5.56–7.77	3.0	0–10	77.6 (38)	1, 2, 3, 4
CR of Inseminators, %	8.0	4.84–7.11	9.0	0–10	77.6 (38)	1, 2, 3, 4
First service CR, %	9.0	6.56–7.90	3.0	0–10	59.2 (29)	1, 2, 4
Age at first service, d	8.0	5.39–7.66	7.5	0–10	59.2 (29)	1, 2, 4
Heifers culled for reproductive reasons, %	8.0	5.23–7.54	8.5	0–10	59.2 (29)	1, 2, 4

Results are expressed as median, CI 95%, interquartile range, minimum (Min) and maximum (Max) values, and proportion of consultants that rated the parameter as highly important (number of observations within brackets). The clusters obtained after hierarchical clustering analysis where most of these highly important parameters were present are also depicted. Parameters are sorted according to the proportion of consultants that rated the parameter as highly important, from highest to lowest.

CI, Confidence interval 95%; CR, Conception rate.

In agreement with Section 3 (Cows' reproduction), the consultants are very aware to continuously analyze data related to the probability of getting heifers pregnant and having first calving at the right time. More than 50% of the consultants surveyed also want to continuously monitor the percentage of heifers culled for reproductive reasons as a primary cause. A myriad of different diseases, syndromes, or events can cause culling due to reproductive reasons and it seems that consultants do not prioritize to go deep into what is causing this type of culling, until data show an alarm. Otherwise, consultants would have higher rated items that could be a direct cause of culling due to reproductive reasons, such as % of anovulatory heifers, % pregnancy loss, and abortion >90 days (all of them rated with a median of 6—moderately important-) or % of ovarian cysts (rated with a 0, as irrelevant).

As mentioned in a companion study (22), authors have carried out research to decipher if consultants specialized in dairy reproduction use the same KPI for two different scenarios: first visit and routine visits. Although some of the KPI are present in both scenarios, some remarkable differences are present. As an example, postpartum and metabolic diseases were not considered to be highly important to analyze the reproductive performance of a farm in a first visit; while metritis, hypocalcaemia, ketosis, and retained placenta are considered critical to monitor by more than 50% of the consultants surveyed during routine visits in the farm. Another interesting difference between the two scenarios is the distributions of consultants among the different clusters in each section related to the years of experience and/or size of the farm with the pattern of response to the questions. The same consultants surveyed for both scenarios, when surveyed for a first visit scenario showed significant different distribution among clusters in Sections 2 and 4 (general data and postpartum and metabolic diseases, respectively) for both years of experience and size of the farm, and in Section 3 (Cows' reproduction) for size of the farm. In case of the answers related to routine visits, there were no differences in the Chi square test between neither the years of experience nor the size of the farm and the pattern in the answers to the questions. One logical explanation to this would be that part of the consultants surveyed, do not differentiate between the two scenarios proposed and would use the same KPI in both situations. Finally, consultants rated different numbers of parameters to be highly important KPI to assess reproductive performance in a farm. When consultants assess for a first visit, 27 parameters were considered as highly important in contrast to the 34 parameters considered for routine visits.

## 4. Conclusion

Consultants specialized in cow and heifer reproduction use several KPI to evaluate different sections or areas that directly or indirectly affect cattle reproduction, such as production parameters, reproduction parameters that reflect biological, management and economic factors, transition period, and heifer rearing process. Consultants are aware of using KPI that reflect heat detection, fertility, and farming efficiency. In addition, the KPI also include those that can provide information on reproductive efficiency in the near future for cows, such as postpartum and metabolic diseases. However, parameters that are relatively old and have been shown to be ineffective, in terms of reproductive performance control, are still highly regarded by a majority of consultants during routine visit scenarios.

From the 178 parameters presented to be KPI to assess the reproductive performance of a dairy farm by routine visits, 34 were considered as primary KPI by a majority of the consultants:

General data of the farm: pregnant cows (%), cows culled for reproductive reasons (%), average DIM (d), and replacement (%).

Cow reproduction: first service CR (%), overall pregnancy rate (%), CR (%), heat detection rate (%), days open (n), abortion >90 days (%), CR at first service in first lactation cows (%), 18–24 days return to service/heat (%), interval service to service (d), calving to first service interval (d), CR first service in multiparous cows (%), conceiving of served (%), 21 days pregnancy rate (%), pregnancy loss (%), calvings per month (*n*), services per pregnancy (*n*), CR of multiparous cows (%), CR of inseminators (%), and CR synchronized cows (%).

Postpartum and metabolic disease: Metritis (%), hypocalcaemia (%), clinical and subclinical ketosis (%), and retained placenta (%).

Heifer reproduction: age at first calving (d), CR (%), heat detection rate (%), CR of inseminators (%), first service CR (%), age at first service (d), and heifers culled for reproductive reasons (%).

The parameters rated with the highest importance (rate 10) that could be considered for an easy, fast, and universal use during routine visits to assess the reproductive status were: First service CR (%), overall pregnancy rate (%) of cows, and age at first calving (d) for heifers.

Neither the farm size nor the years of experience of the consultants are factors influencing the type and number of parameters chosen to be KPI during routine visits to assess reproductive performance.

## Data availability statement

The original contributions presented in the study are included in the article/Supplementary material, further inquiries can be directed to the corresponding author.

## Ethics statement

The study was reviewed and approved by Ethic Committee of the University of Lleida. Written informed consent from the participants was not required to participate in this study in accordance with the national legislation and the institutional requirements.

## Author contributions

RA: conceptualization, methodology, investigation, writing—original draft, and visualization. LF: formal analysis, methodology, validation, writing—original draft, and visualization. AB: methodology, validation, visualization, and writing—review and editing. All authors contributed to the article and approved the submitted version.
