# Supplementary material for: Key performance indicators used by dairy consultants during the evaluation of reproductive performance during routine visits
Source: Front Vet Sci. 2023 Jun 2;10:1165184. doi: 10.3389/fvets.2023.1165184 (PMC10272744; doi:10.3389/fvets.2023.1165184)
Supplement: Supplementary file 1 [file Data_Sheet_1.PDF]

Supplementary Table 1. Content of the survey sent in Google Forms format to the participants. The table includes the section, the parameter, the exact definition and the type of answer that consultants had to fulfill.

SECTION	PARAMETER	DEFINITION	ANSWEAR
Section 1 Consultant and farm model			
	Country	Country were the consultant mostly works	Text
	Age	Age of the consultant	20 to 30<br>31 to 40<br>41 to 50<br>51 to 60<br>61 to 70
	Years of Experience	Years of experience consulting for reproduction in dairy farms	0 to 5<br>5 to 10<br>10 to 15<br>15 to 20<br>20 to 25<br>>25
	Farm System	Most common farm system that consultant advises	Pasture at any time<br>Never Pastures
	Cows Breed	Most common breed present in the farms that consultant advises	Text
	Main Breeding system	Most common breeding technique used in the farms that consultant advises	Mating<br>Artificial Insemination<br>Embryo transfer<br>In vitro fertilization
	Calving Season	Farms with the goal of having one or two calving seasons or not (homogeneous number of calvings during the year)	Yes<br>No
	Type of stall	Type of allocation that cows have in the stall	Tied<br>Cubicles/Free Stall<br>Bedded Pack
	Number of milkings/ day	Number of times that cows are milked	2<br>3
	Size of the Farm	Numbers of milking cows that farms advised have.	1 to 50

			51-150<br>151-300<br>301-500<br>501-700<br>701-1000<br>1001-3000<br>>3000
	Close up pen		Yes<br>No
	Time span of data requested	Period of time that data requested by the consultant in the first visit to the farm would cover.	Current Year<br>Last 3 months<br>Last 6 months<br>Last year<br>Last 3 years<br>Last 5 years<br>Last 10 years
Section 2 General data of the farm			
	Average number of lactation	Average of all lactation numbers of cows during the period	0 1 2 3 4 5 6 7 8 9 10
	Total number of cows	Average number of cows present in the farm during the period	0 1 2 3 4 5 6 7 8 9 10
	Number of milking cows	Average number of milking cows during the period	0 1 2 3 4 5 6 7 8 9 10
	Number of 1st lactation cows	Average number of 1st lactation milking cows during the period	0 1 2 3 4 5 6 7 8 9 10
	% 1st lactation cows	Average number of 1st lactation milking cows divided by the total number of milking cows during the period	0 1 2 3 4 5 6 7 8 9 10
	% Dry cows	Average number of dry off cows divided by the total number of cows present in the farm during the period	0 1 2 3 4 5 6 7 8 9 10
	Number of dry cows	Average number of cows that are dry during the period	0 1 2 3 4 5 6 7 8 9 10
	Monthly milk yield	Average production of kilos of milk produced every month	0 1 2 3 4 5 6 7 8 9 10

	Daily milk yield	Average production of kilos of milk produced daily	0 1 2 3 4 5 6 7 8 9 10
	Lactating cows daily milk yield	Average of the total kilos of milk produced in one day divided by for the number of milking cows	0 1 2 3 4 5 6 7 8 9 10
	All cows daily milk yield	Average of the total kilos of milk produced in one day divided by the total (both milking and dry) number of cows present in the farm	0 1 2 3 4 5 6 7 8 9 10
	Total number of pregnant cows	Average total number of pregnant cows during the period	0 1 2 3 4 5 6 7 8 9 10
	% of pregnant cows	Average total number of pregnant cows divided by the total number of cows present in the farm during the period	0 1 2 3 4 5 6 7 8 9 10
	Days in milk (DIM)	Average days in milk for all cows during the period	0 1 2 3 4 5 6 7 8 9 10
	Average days dry	Average days in dry off for all cows during the period	0 1 2 3 4 5 6 7 8 9 10
	305 day yield	Average cumulative production by day 305 of lactation for all cows during the period.	0 1 2 3 4 5 6 7 8 9 10
	Culling rate	Average number of cows culled, sold, dead or transferred out of the herd divided by the average number of total cows (both milking and dry) in the same period.	0 1 2 3 4 5 6 7 8 9 10
	Failure to conceive culling rate	Average number of cows culled due to a failure to conceive as a percentage of the total number of eligible cows calving in the period.	0 1 2 3 4 5 6 7 8 9 10
	% "do not breed" cows	Average number of cows with decision of "do not breed" divided by the total number of cows in the same period	0 1 2 3 4 5 6 7 8 9 10
	% Cows culled for reproductive reason	Average number culled cows for reproductive reasons divided by the total number cows culled during the period	0 1 2 3 4 5 6 7 8 9 10
	% Cows culled for lameness reason	Average number culled cows for lameness reasons divided by the total number cows culled during the period	0 1 2 3 4 5 6 7 8 9 10
	% Cows culled for mastitis reason	Average number culled cows for mastitis reasons divided by the total number cows culled during the period	0 1 2 3 4 5 6 7 8 9 10
	% Cows culled for accident reason	Average number culled cows due to accidents	0 1 2 3 4 5 6 7 8 9 10

		divided by the total number cows culled during the period	
	Average somatic cell count (SCC)	Average SCC of all milk samples taken in the last 12 months.	0 1 2 3 4 5 6 7 8 9 10
	% Clinical mastitis	Average number of clinical mastitis cases divided by the total number cows lactation during the period	0 1 2 3 4 5 6 7 8 9 10
	% Lameness	Average number of lameness cases divided by the total number of cows (both milking and dry) during the period	0 1 2 3 4 5 6 7 8 9 10
	Peak milk 1st lactation cows	Average of highest level of milk in kg that 1st lactation cows produce during the 1st lactation.	0 1 2 3 4 5 6 7 8 9 10
	Peak milk 2nd lactation cows	Average of highest level of milk in kg that 2nd lactation cows produce during the 2nd lactation.	0 1 2 3 4 5 6 7 8 9 10
	Peak milk 3rd lactation cows	Average of highest level of milk in kg that 3rd lactation cows produce during the 3rd lactation.	0 1 2 3 4 5 6 7 8 9 10
	Peak milk >3rd lactation cows	Average of highest level of milk in kg that cows with more than three lactations produce during their present lactation.	0 1 2 3 4 5 6 7 8 9 10
	Peak milk (DIM) 1st lactation cows	Average of days in milk that 1st lactation cows had to reach peak milk.	0 1 2 3 4 5 6 7 8 9 10
	Peak milk (DIM) 2nd lactation cows	Average of days in milk that 2nd lactation cows had to reach the peak milk.	0 1 2 3 4 5 6 7 8 9 10
	Peak milk (DIM) 3rd lactation cows	Average of days in milk that 3rd lactation cows had to reach the peak milk.	0 1 2 3 4 5 6 7 8 9 10
	Peak milk (DIM) >3rd lactation cows	Average of days in milk that cows with more than three lactations had to reach the peak milk.	0 1 2 3 4 5 6 7 8 9 10
	% Replacement	Average number of heifers or purchased cows that go into "milking cow" status for the first time in that herd divided for the total number of cows (both lactating and dry) during the period	0 1 2 3 4 5 6 7 8 9 10
	Average lactation number of culled cows	Average lactation number of cows culled in the farm during the period	0 1 2 3 4 5 6 7 8 9 10
	Total number of cows culled	Average number of cows culled in the farm during the period	0 1 2 3 4 5 6 7 8 9 10
	Average DIM culled cows	Average days in milk for all cows culled during the	0 1 2 3 4 5 6 7 8 9 10

		period	
	Herd status for Brucellosis	Herd status (Positivity and/or Prevalence) for *Brucella spp*	Present<br>Absent
	Herd status for Neosporosis	Herd status (Positivity and/or Prevalence) for *Neospora caninum*	Present<br>Absent
	Herd status for BVDV	Herd status (Positivity and/or Prevalence) for Bovine Viral Diarrhoea Virus	Present<br>Absent
	Herd status for IBR-IPV	Herd status (Positivity and or Prevalence) for Infectious Bovine Infectious Rhinotracheitis-Infectious Pustular Vulvovaginitis-Infectious Balanitis caused by Bovine Herpesvirus	Present<br>Absent
	Herd status for FMD	Herd status (Positivity and/or Prevalence) for Foot and Mouth Disease caused by Aftovirus	Present<br>Absent
	Other parameters useful for you	Other parameters that the consultant uses and not included in the survey	Text
Section 3 Cow reproduction			
	Voluntary waiting period	Average number of postpartum days that cows are deliberately left unserved	0 1 2 3 4 5 6 7 8 9 10
	% conceiving of served	Average proportion of the total number of animals served that conceive	0 1 2 3 4 5 6 7 8 9 10
	Overall pregnancy rate	Average number of pregnancies divided by the total number of eligible services	0 1 2 3 4 5 6 7 8 9 10
	First service conception rate	Average number of pregnancies divided by the total number of first services.	0 1 2 3 4 5 6 7 8 9 10
	Days open	Average number of days from calving to conception for those cows conceiving and from calving to culling for those failing to conceive	0 1 2 3 4 5 6 7 8 9 10
	Days to culling	Average number of day between conception and culling	0 1 2 3 4 5 6 7 8 9 10
	Non- return rate	Average number of cows not returning to service as a percentage of the total number of cows served	0 1 2 3 4 5 6 7 8 9 10
	Conception rate	Average number of pregnancies divided by the total number of services	0 1 2 3 4 5 6 7 8 9 10
	% Ovarian cysts	Average number of cows diagnosed with an ovarian cyst divided by the total number of eligible cows in the herd during the period	0 1 2 3 4 5 6 7 8 9 10

	% Anovulatory cows	Average number of cows diagnosed as anovulatory divided by the total number of eligible cows during the period	0 1 2 3 4 5 6 7 8 9 10
	21d Pregnancy rate	Average number of pregnancies divided by the number of eligible cycles over a 21 days during the evaluation period	0 1 2 3 4 5 6 7 8 9 10
	Services per pregnancy	Average total number of services over the total number of pregnant cows during the period	0 1 2 3 4 5 6 7 8 9 10
	% Pregnancy loss	Average number of pregnant cows that lost pregnancy divided by the total number of pregnant cows during the period	0 1 2 3 4 5 6 7 8 9 10
	% Early pregnancy loss (1-42 days)	Average number of pregnant cows that lost pregnancy during first 42 days of gestation divided by the total number of pregnant during the period	0 1 2 3 4 5 6 7 8 9 10
	% Pregnancy loss(1-90days)	Average number of pregnant cows that lost pregnancy during the first 90 d of gestation divided by the total number of pregnancies during the period	0 1 2 3 4 5 6 7 8 9 10
	% Abortion >90 days	Average number of pregnant cows that lost pregnancy beyond 90 d of gestation divided by the total number of pregnant cows during the period	0 1 2 3 4 5 6 7 8 9 10
	% Conception rate synchronized cows	Average number of pregnant cows from one hormonal treatment divided by the total number of services conducted after a hormonal treatment	0 1 2 3 4 5 6 7 8 9 10
	Conception rate of the sire	Average number of pregnant cows from one sire divided by the total number of services of the same sire during the period	0 1 2 3 4 5 6 7 8 9 10
	Conception rate of inseminators	Average number of pregnant cows serviced by one technician divided by the total number of services that the same technician performed during the period	0 1 2 3 4 5 6 7 8 9 10
	Days at pregnancy diagnosis	Average interval (in days) between service and pregnancy diagnosis	0 1 2 3 4 5 6 7 8 9 10
	% Conception rate first service in 1st lactation cows	Average number of 1st lactation cows pregnant at first service divided by the total number of 1st lactation cows that were first serviced during the	0 1 2 3 4 5 6 7 8 9 10

		period	
	% Conception rate first service in 2nd lactation cows	Average number of 2nd lactation cows pregnant at first service divided by the total number of 2nd lactation cows that were first serviced during the period	0 1 2 3 4 5 6 7 8 9 10
	% Conception rate first service in 3rd lactation cows	Average number of 3rd lactation cows pregnant at first service divided by the total number of 3rd lactation cows that were first serviced during the period	0 1 2 3 4 5 6 7 8 9 10
	% Conception rate first service in >3rd lactation cows	Average number of cows with more than three lactations pregnant at first service divided by the total number cows with more than lactations first serviced during the period	0 1 2 3 4 5 6 7 8 9 10
	% Conception rate first service in multiparous cows	Average number of multiparous cows pregnant at first service divided by the total number of multiparous cows first serviced during the period	0 1 2 3 4 5 6 7 8 9 10
	% Conception rate 1st lactation cows	Average number of 1st lactation cows pregnant divided by the total number 1st lactation cows of serviced during the period	0 1 2 3 4 5 6 7 8 9 10
	% Conception rate 2nd lactation cows	Average number of 2nd lactation cows pregnant divided by the total number of 2nd lactation cows serviced during the period	0 1 2 3 4 5 6 7 8 9 10
	% Conception rate 3rd lactation cows	Average number of 3rd lactation cows pregnant divided by the total number of 3rd lactation cows serviced during the period	0 1 2 3 4 5 6 7 8 9 10
	% Conception rate >3rd lactation cows	Average number of cows with more than three lactations pregnant divided by the total number of cows with more than three lactations serviced during the period	0 1 2 3 4 5 6 7 8 9 10
	% Conception rate multiparous cows	Average number of multiparous cows pregnant divided by the total number multiparous cows serviced during the period	0 1 2 3 4 5 6 7 8 9 10
	Submission rate first 3 weeks	Average percentage of cows receiving at least one insemination during the first three weeks of the breeding period – which begins once the Voluntary Waiting Period has been completed.	0 1 2 3 4 5 6 7 8 9 10

	Submission rate	Average number of cows in the herd receiving a first service	0 1 2 3 4 5 6 7 8 9 10
	Calving to first service interval	Average interval (in days) between calving and first service	0 1 2 3 4 5 6 7 8 9 10
	Interval heat to heat	Average number of days between consecutive heats	0 1 2 3 4 5 6 7 8 9 10
	Interval service to service	Average number of days between consecutive services	0 1 2 3 4 5 6 7 8 9 10
	Heat detection rate	Average number of heats identified divided by the number of eligible cycles during an eligible period	0 1 2 3 4 5 6 7 8 9 10
	2–17 day return to service/heat, %	Average proportion of eligible cows that return to estrous within 2 to 17 days	0 1 2 3 4 5 6 7 8 9 10
	18–24 day return to service/heat, %	Average proportion of eligible cows that return to estrous within 18 to 24 days	0 1 2 3 4 5 6 7 8 9 10
	25–35 day return to service/heat, %	Average proportion of eligible cows that return to estrous within 25 to 35 days	0 1 2 3 4 5 6 7 8 9 10
	36–48 day return to service/heat, %	Average proportion of eligible cows that return to estrous within 36 to 48 days	0 1 2 3 4 5 6 7 8 9 10
	>49 day return to service/heat, %	Average proportion of eligible cows that return to estrous after 49 days	0 1 2 3 4 5 6 7 8 9 10
	100-Day In-calf rate	Average percentage of cows in the herd confirmed pregnant within 100 days of calving	0 1 2 3 4 5 6 7 8 9 10
	% Cows served <90 DIM	Average proportion of cows that are served before 90 days in milk	0 1 2 3 4 5 6 7 8 9 10
	Herd calving to conception interval	Average number of days from calving to the service at which a cow becomes pregnant	0 1 2 3 4 5 6 7 8 9 10
	Calving interval	Average number of days elapsed between current and previous calving	0 1 2 3 4 5 6 7 8 9 10
	% Cows not pregnant >200 DIM	Average proportion of cows that are not pregnant after day 200 in milk	0 1 2 3 4 5 6 7 8 9 10
	% Cows not pregnant >150 DIM	Average proportion of cows that are not pregnant after day 150 in milk	0 1 2 3 4 5 6 7 8 9 10
	Calvings per month	Average of the number of calvings every month	0 1 2 3 4 5 6 7 8 9 10
	% 1st lactation cows calved	Average number of heifers that calved divided by the total number of cows that calved	0 1 2 3 4 5 6 7 8 9 10
	OTHER PARAMETERS USEFUL FOR YOU	Other parameters that the consultant uses and not included in the survey	Text

Section 4 Postpartum and metabolic diseases			
	% Metritis	Average number of cows with metritis divided by the total number of cows that calved during the period	0 1 2 3 4 5 6 7 8 9 10
	% Retained placenta	Average number of cows with retained placenta divided by the total number of cows that calved during the period	0 1 2 3 4 5 6 7 8 9 10
	% Clinical ketosis	Average number of cows with clinical ketosis divided by the total number of cows that calved during the period	0 1 2 3 4 5 6 7 8 9 10
	% Clinical and subclinical ketosis	Average number of cows with subclinical and clinical ketosis divided by the total number of cows that calved during the period	0 1 2 3 4 5 6 7 8 9 10
	% Hypocalcaemia	Average number of cows with hypocalcaemia divided by the total number of cows that calved during the period	0 1 2 3 4 5 6 7 8 9 10
	% Stillbirth	Average number of cows that delivered a dead calf or the calf died within the first 24 hours divided by the total number of cows that calved during the period	0 1 2 3 4 5 6 7 8 9 10
	% Twins	Average number of cows with twins parturition divided by the total number of cows that calved during the period	0 1 2 3 4 5 6 7 8 9 10
	% Dystocia	Average number of cows with dystocia divided by the total number of cows that calved	0 1 2 3 4 5 6 7 8 9 10
	% Metritis 1st lactation cows	Average number of 1st lactation cows with metritis divided by the total number of 1st lactation cows that calved	0 1 2 3 4 5 6 7 8 9 10
	% Retained placenta 1st lactation cows	Average number of 1st lactation cows with retained placenta divided by the total number of 1st lactation cows that calved	0 1 2 3 4 5 6 7 8 9 10
	% Clinical ketosis 1st lactation cows	Average number of 1st lactation cows with clinical ketosis divided by the total number of 1st lactation cows that calved	0 1 2 3 4 5 6 7 8 9 10
	% Clinical and subclinical ketosis 1st lactation cows	Average number of 1st lactation cows with	0 1 2 3 4 5 6 7 8 9 10

		subclinical and clinical ketosis divided by the total number of 1st lactation cows that calved	
	% Hypocalcaemia 1st lactation cows	Average number of 1st lactation cows with hypocalcaemia divided by the total number of 1st lactation cows that calved	0 1 2 3 4 5 6 7 8 9 10
	% Stillbirth 1st lactation cows	Average number of 1st lactation cows that delivered a dead calf or the calf died within the first 24 hours divided by the total number of 1st lactation cows that calved	0 1 2 3 4 5 6 7 8 9 10
	% Twins 1st lactation cows	Average number of 1st lactation cows with twins parturition divided by the total number of 1st lactation cows that calved	0 1 2 3 4 5 6 7 8 9 10
	% Dystocia 1st lactation cows	Average number of 1st lactation cows with dystocia divided by the total number of 1st lactation cows that calved	0 1 2 3 4 5 6 7 8 9 10
	% Metritis multiparous	Average number of multiparous cows with metritis divided by the total number of multiparous cows that calved	0 1 2 3 4 5 6 7 8 9 10
	% Retained placenta multiparous	Average number of multiparous cows with retained placenta divided by the total number of multiparous cows that calved	0 1 2 3 4 5 6 7 8 9 10
	% Clinical ketosis multiparous	Average number of multiparous cows with clinical ketosis divided by the total number of multiparous cows that calved	0 1 2 3 4 5 6 7 8 9 10
	% Clinical and subclinical ketosis multiparous	Average number of multiparous cows with subclinical and clinical ketosis divided by the total number of multiparous cows that calved	0 1 2 3 4 5 6 7 8 9 10
	% Hypocalcaemia multiparous	Average number of multiparous cows with hypocalcaemia divided by the total number of multiparous cows that calved	0 1 2 3 4 5 6 7 8 9 10
	% Stillbirth multiparous	Average number of multiparous cows that delivered a dead calf or the calf died within the first 24 hours divided by the total number of multiparous cows that calved	0 1 2 3 4 5 6 7 8 9 10
	% Twins multiparous	Average number of multiparous cows with twins parturition divided by the total number of	0 1 2 3 4 5 6 7 8 9 10

		multiparous cows that calved	
	% Dystocia multiparous	Average number of multiparous cows with dystocia divided by the total number of multiparous cows that calved	0 1 2 3 4 5 6 7 8 9 10
	% Incorrect uterine involution after 30DIM	Average number of cows with an incorrect uterine involution after day thirty in milk divided by the total number of cows that calved	0 1 2 3 4 5 6 7 8 9 10
	% Incorrect uterine involution after 30DIM 1st lactation cows	Average number of 1st lactation cows with an incorrect uterine involution after day thirty in milk divided by the total number of 1st lactation cows that calved	0 1 2 3 4 5 6 7 8 9 10
	% Incorrect uterine involution after 30DIM multiparous	Average number of multiparous cows with an incorrect uterine involution after day thirty in milk divided by the total number of multiparous cows that calved	0 1 2 3 4 5 6 7 8 9 10
	% Pyometra	Average number of cows with pyometra divided by the total number of cows that calved	0 1 2 3 4 5 6 7 8 9 10
	% Pyometra 1st lactation	Average number of 1st lactation cows with pyometra divided by the total number of 1st lactation cows that calved	0 1 2 3 4 5 6 7 8 9 10
	% Pyometra multiparous	Average number of multiparous cows with pyometra divided by the total number of multiparous cows that calved	0 1 2 3 4 5 6 7 8 9 10
	% Perineal injury	Average number of cows with perineal injury divided by the total number of cows that calved	0 1 2 3 4 5 6 7 8 9 10
	% Perineal injury 1st lactation	Average number of 1st lactation cows with perineal injury divided by the total number of 1st lactation cows that calved	0 1 2 3 4 5 6 7 8 9 10
	% Perineal injury multiparous	Average number of multiparous cows with perineal injury divided by the total number of multiparous cows that calved	0 1 2 3 4 5 6 7 8 9 10
	% Abomasal pathology	Average number of cows diagnosed with abomasal pathology divided by the total number of cows calved	0 1 2 3 4 5 6 7 8 9 10
	% Abomasal pathology 1st lactation	Average number of 1st lactation cows diagnosed with abomasal pathology divided by the total	0 1 2 3 4 5 6 7 8 9 10

		number of 1st lactation cows calved	
	% Abomasal pathology multiparous	Average number of multiparous cows diagnosed with abomasal pathology divided by the total number of multiparous cows calved	0 1 2 3 4 5 6 7 8 9 10
	OTHER PARAMETERS USEFUL FOR YOU	Other parameters that the consultant uses and not included in the survey	Text
Section 5 Heifer reproduction			
	Conception rate	Average number of pregnancies in heifers divided by the total number of services in heifers during the period	0 1 2 3 4 5 6 7 8 9 10
	First service conception rate	Average number of heifers pregnant at first service divided by the total number of heifers that were first serviced during the period	0 1 2 3 4 5 6 7 8 9 10
	Interval heat to heat	Average number of days between consecutive heats	0 1 2 3 4 5 6 7 8 9 10
	Interval service to service	Average number of days between consecutive services	0 1 2 3 4 5 6 7 8 9 10
	Heat detection rate	Average number of heats identified divided by the number of eligible cycles during the period	0 1 2 3 4 5 6 7 8 9 10
	% Ovarian Cysts	Average number of eligible heifers diagnosed with an ovarian cyst divided by the total number of eligible heifers to be serviced in the herd.	0 1 2 3 4 5 6 7 8 9 10
	% Anovulatory heifers	Average number of eligible heifers diagnosed as anovulatory divided by the total number of eligible heifers to be serviced in the herd	0 1 2 3 4 5 6 7 8 9 10
	21d Pregnancy rate	Average number of heifers that became pregnant divided by the number of heifers eligible over a 21-day period	0 1 2 3 4 5 6 7 8 9 10
	Services per pregnant heifer	Average number of services in heifers over the total number of pregnant heifers during the period	0 1 2 3 4 5 6 7 8 9 10
	Culling rate heifers	Average number of heifers that are sold, die or are transferred out of the herd before having the first parturition in the period divided by the total number of heifers	0 1 2 3 4 5 6 7 8 9 10
	% Heifers culled for reproductive reason	Average number culled heifers for reproductive reasons divided by the total number heifers culled	0 1 2 3 4 5 6 7 8 9 10

	% "do not breed" heifers	Average number of heifers with decision of "do not breed" divided by the total number of heifers	0 1 2 3 4 5 6 7 8 9 10
	Age at first service	Average age at which heifers were serviced for the first time	0 1 2 3 4 5 6 7 8 9 10
	Age at first calving	Average age at which heifers calved for the first time	0 1 2 3 4 5 6 7 8 9 10
	% Heifers calving <24 months old	Average proportion of heifers that calved younger than 24 months	0 1 2 3 4 5 6 7 8 9 10
	% Heifers calving <23 months old	Average proportion of heifers that calved younger than 23 months	0 1 2 3 4 5 6 7 8 9 10
	% Heifers calving <22 months old	Average proportion of heifers that calved younger than 22 months	0 1 2 3 4 5 6 7 8 9 10
	% Heifers calving <21 months old	Average proportion of heifers that calved younger than 21 months	0 1 2 3 4 5 6 7 8 9 10
	Conception rate synchronized heifers	Average number of pregnant heifers from one hormonal treatment divided by the number of services in heifers using a hormonal treatment.	0 1 2 3 4 5 6 7 8 9 10
	Conception rate of the sire	Average number of pregnant heifers from one sire divided by the total number of services of the same sire.	0 1 2 3 4 5 6 7 8 9 10
	Conception rate of inseminators	Average number of pregnant heifers from one technician divided by the total number of services performed by that technician	0 1 2 3 4 5 6 7 8 9 10
	Days at pregnancy diagnosis	Average interval (in days) between service and pregnancy diagnosis	0 1 2 3 4 5 6 7 8 9 10
	Number of heifers	Average number of heifers present in the herd	0 1 2 3 4 5 6 7 8 9 10
	% heifers/cows	Average number of heifers divided by the number of cows present in the herd	0 1 2 3 4 5 6 7 8 9 10
	% of heifers <14 months old	Average proportion (over total number of heifers) of heifers younger than 14 months	0 1 2 3 4 5 6 7 8 9 10
	% of heifers >14 months old	Average proportion (over total number of heifers) older than 14 months	0 1 2 3 4 5 6 7 8 9 10
	% of heifers <13 months old	Average proportion (over total number of heifers) of heifers younger than 13 months	0 1 2 3 4 5 6 7 8 9 10
	% of heifers >13 months old	Average proportion (over total number of heifers) of heifers older than 13 months	0 1 2 3 4 5 6 7 8 9 10

	% of heifers <12 months old	Average proportion (over total number of heifers) of heifers younger than 12 months	0 1 2 3 4 5 6 7 8 9 10
	% of heifers >12 months old	Average proportion (over total number of heifers) of heifers older than 12 months	0 1 2 3 4 5 6 7 8 9 10
	% of heifers <11 months old	Average proportion (over total number of heifers) of heifers younger than 11 months	0 1 2 3 4 5 6 7 8 9 10
	% of heifers >11 months old	Average proportion (over total number of heifers) of heifers older than 11 months	0 1 2 3 4 5 6 7 8 9 10
	% Heifers >14 months old not serviced	Average proportion of heifers older than 14 months that are not served	0 1 2 3 4 5 6 7 8 9 10
	% Heifers >13 months old not serviced	Average proportion of heifers older than 13 months that are not served	0 1 2 3 4 5 6 7 8 9 10
	% Heifers >12 months old not serviced	Average proportion of heifers older than 12 months that are not served	0 1 2 3 4 5 6 7 8 9 10
	% Heifers >11 months old not serviced	Average proportion of heifers older than 11 months that are not served	0 1 2 3 4 5 6 7 8 9 10
	% Heifers pregnant	Average proportion of heifers that are pregnant	0 1 2 3 4 5 6 7 8 9 10
	% Pregnancy loss	Average number of pregnant heifers that lost pregnancy divided by the total number of pregnant heifers	0 1 2 3 4 5 6 7 8 9 10
	% Early pregnancy loss (1-42 days)	Average number pregnant heifers that lost 1-42 days pregnancy divided by the total number of pregnant heifers	0 1 2 3 4 5 6 7 8 9 10
	% Pregnancy loss(1-90days)	Average number pregnant heifers that lost 1-90 days pregnancy divided by the total number of pregnant heifers	0 1 2 3 4 5 6 7 8 9 10
	% Abortion >90 days	Average number pregnant heifers that lost more than 90 days pregnancies divided by the total number of pregnant heifers	0 1 2 3 4 5 6 7 8 9 10
	% of open heifers > 12 months	Average proportion of heifers older than 12 months that are not pregnant	0 1 2 3 4 5 6 7 8 9 10
	% of open heifers > 13 months	Average proportion of heifers older than 13 months that are not pregnant	0 1 2 3 4 5 6 7 8 9 10
	% of open heifers > 14 months	Average proportion of heifers older than 14 months that are not pregnant	0 1 2 3 4 5 6 7 8 9 10
	% of open heifers > 15 months	Average proportion of heifers older than 15 months	0 1 2 3 4 5 6 7 8 9 10

		that are not pregnant	
	% of open heifers > 16 months	Average proportion of heifers older than 16 months that are not pregnant	0 1 2 3 4 5 6 7 8 9 10
	% of open heifers > 17 months	Average proportion of heifers older than 17 months that are not pregnant	0 1 2 3 4 5 6 7 8 9 10
	% of heifers <2 standard deviations from 400 kg at 400d	Average proportion of heifers with 2 standard deviations below 400 kg at 400 d	0 1 2 3 4 5 6 7 8 9 10
	% of heifers < 580 kg at calving	Average proportion of heifers calving with less than 580 kg	0 1 2 3 4 5 6 7 8 9 10
	Heifer efficiency, %	Average proportion of heifers that calved below or at 24 months of age divided by the total number of heifers that were born during that period	0 1 2 3 4 5 6 7 8 9 10
	OTHER PARAMETERS USEFUL FOR YOU	Other parameters that the consultant uses and not included in the survey	Text

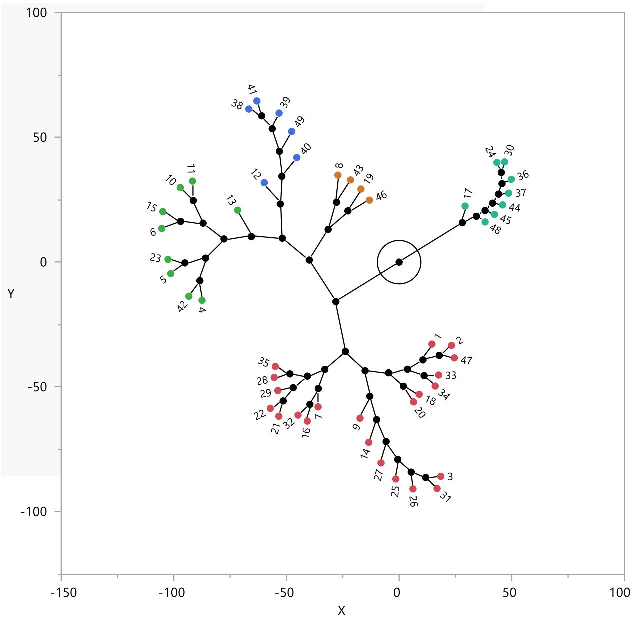

Supplementary Figure 1. Constellation plot showing the results of a hierarchical analysis of the 49 surveys answered of Section 2. General data of the farm. The numeration corresponds to each consultant that answered the survey. Cluster 1, 2, 3, 4 and 5 are represented in red circles, green crosses, blue squares, red crosses and green triangles, respectively.

Supplementary Table 2. Answer pattern for the 5 clusters obtained from the 43 parameters evaluated in the Section 2. General data of the farm after hierarchical clustering analysis. It is detailed in brackets the number of surveys answered belonging to each cluster. Parameters considered as highly important, moderately important, low important and irrelevant are showed in green, blue, orange and red, respectively.

General Data of the Farm	Cluster 1 (22)	Cluster 2 (9)	Cluster 3 (6)	Cluster 4 (4)	Cluster 5 (8)
Average number of lactations, n	blue	blue	orange	orange	red
Total number of cows, n	blue	blue	orange	red	red
Number of milking cows, n	blue	green	orange	green	red
Number of 1st lact cows, n	green	blue	orange	orange	red
1st lact cows, %	green	blue	blue	red	red
Dry cows, %	green	blue	blue	red	red
Number of dry cows, n	blue	blue	orange	red	red
Monthly milk yield, kg	blue	orange	orange	orange	red
Daily milk yield, kg	blue	blue	orange	orange	red
Lactating cows daily milk yield, kg	blue	green	blue	orange	red
All cows daily milk yield, kg	blue	orange	orange	orange	red
Total pregnant cows, n	blue	red	orange	orange	red
Pregnant cows, %	green	green	green	blue	red

Average DIM, d					
Average days dry, d					
305 day yield, kg					
Culling rate, %					
Failure to conceive culling rate, %					
"Do not breed" cows, %					
Cows culled for reproductive reason, %					
Cows culled for lameness reason, %					
Cows culled for mastitis reason, %					
Cows culled for accident reason, %					
Average SCC, SCC/mL					
Clinical mastitis, %					
Lameness, %					
Peak milk 1st lact cows, kg					
Peak milk 2nd lact cows, kg					
Peak milk 3rd lact cows, kg					
Peak milk >3rd lact cows, kg					
Peak milk 1st lact cows, DIM					
Peak milk 2nd lact cows, DIM					

Peak milk 3rd lact cows, DIM					
Peak milk >3rd lact cows, DIM					
Replacement, %					
Average lact of culled cows, n					
Number of cows culled, n					
DIM culled cows, n					
Herd status for brucellosis, yes/no					
Herd status for neosporosis, yes/no					
Herd status for BVDV, yes/no					
Herd status for IBR-IPV, yes/no					
Herd status for FMD, yes/no					

DIM, Days in milk; lact, lactation; SCC, Somatic Cell Count; BVDV, Bovine Viral Diarrhea Virus; IBR-IPV, Infectious Bovine Rhinotracheitis - Infectious Pustular Vulvovaginitis; FMD, Foot and Mouth Disease.

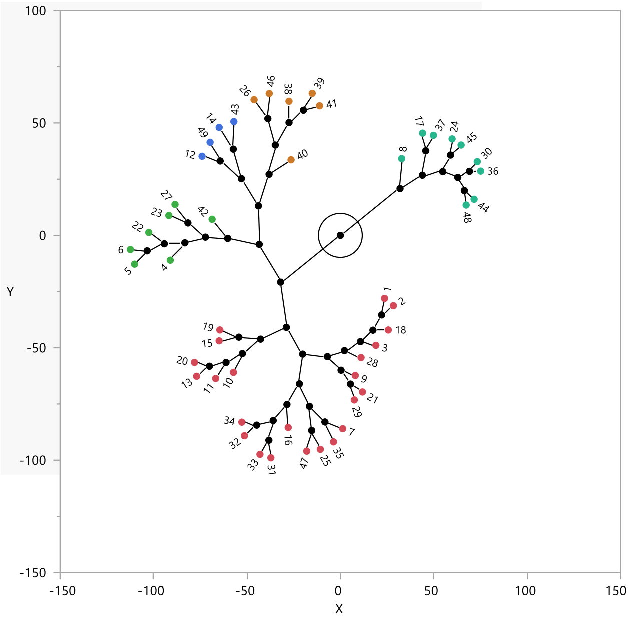

Supplementary Figure 2. Constellation plot showing the results of a hierarchical analysis of the 49 surveys answered of Section 3. Cows' reproduction. The numeration corresponds to each consultant that answered the survey. Cluster 1, 2, 3, 4 and 5 are represented in red, dark green, blue, brown and soft green color, respectively.

Supplementary Table 3. Answer pattern for the 5 clusters obtained from the 49 parameters evaluated in the Section 3. Cows' Reproduction after hierarchical clustering analysis. It is detailed in brackets the number of surveys answered belonging to each cluster. Parameters considered as highly important, moderately important, low important and irrelevant are showed in green, blue, orange and red, respectively.

Cows' Reproduction Parameter	Cluster 1 (23)	Cluster 2 (7)	Cluster 3 (4)	Cluster 4 (6)	Cluster 5 (9)
Voluntary waiting period, d	green	blue	green	blue	orange
Percent conceiving of served, %	green	blue	green	green	red
Overall pregnancy rate, %	green	green	blue	green	orange
First service CR, %	green	green	green	green	red
Days open, n	green	green	orange	green	orange
Days to culling, n	orange	red	orange	orange	red
Non- return rate, %	blue	orange	red	blue	red
CR, %	green	green	blue	green	orange
Ovarian cysts, %	blue	orange	blue	blue	red
Anovulatory cows, %	green	blue	blue	green	red
21d Pregnancy rate, %	green	green	red	blue	red
Services per pregnancy, n	green	green	orange	blue	red
Pregnancy loss, %	green	green	blue	blue	orange
Early pregnancy loss (1-42 days), %	blue	orange	blue	orange	red

Pregnancy loss (1-90days), %					
Abortion >90 days, %					
CR synchronized cows, %					
CR of the sire, %					
CR of inseminators, %					
Days at pregnancy diagnosis, n					
CR first service in 1st lact cows, %					
CR first service in 2nd lact cows, %					
CR first service in 3rd lact cows, %					
CR first service in >3rd lact cows, %					
CR first service in multiparous cows, %					
CR 1st lact cows, %					
CR 2nd lact cows, %					
CR 3rd lact cows, %					
CR >3rd lact cows, %					
CR multiparous cows, %					
Submission rate first 3 weeks, %					
Submission rate, %					
Calving to first service interval, d					

Interval heat to heat, d					
Interval service to service, d					
Heat detection rate, %					
2–17 d. return to service/heat, %					
18–24 d. return to service/heat, %					
25–35 d. return to service/heat, %					
36–48 d. return to service/heat, %					
>49 d. return to service/heat, %					
100-Day In-calf rate, %					
Cows served <90 DIM, %					
Herd calving to conception interval, d					
Calving interval, d					
Cows not pregnant >200 DIM, %					
Cows not pregnant >150 DIM, %					
Calvings per month, n					
1st lact cows calved, %					

CR, Conception rate; DIM, Days in milk; lact, lactation

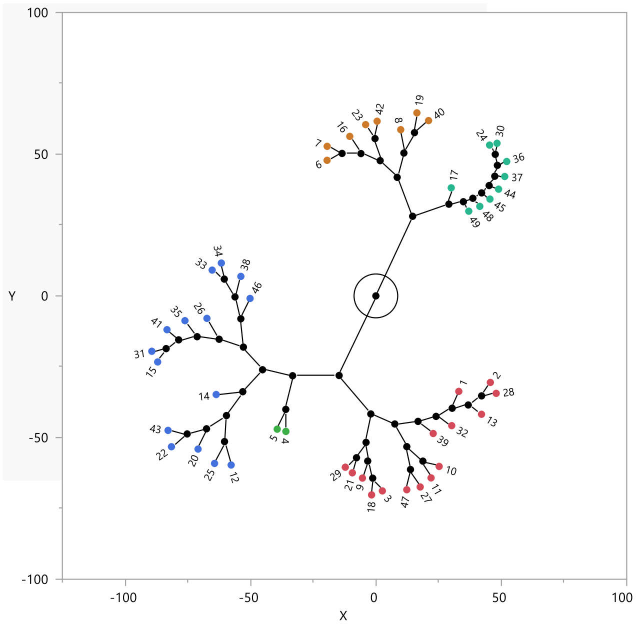

Supplementary Figure 3. Constellation plot showing the results of a hierarchical analysis of the 49 surveys answered of Section 4. Postpartum and metabolic diseases. The numeration corresponds to each consultant that answered the survey. Cluster 1, 2, 3, 4 and 5 are represented in red, dark green, blue, brown and soft green color, respectively.

Supplementary Table 4. Answer pattern for the 5 clusters obtained from the 36 parameters evaluated in the Section 4. Postpartum and metabolic diseases after hierarchical clustering analysis. It is detailed in brackets the number of surveys answered belonging to each cluster. Parameters considered as highly important, moderately important, low important and irrelevant are showed in green, blue, orange and red, respectively.

Postpartum and metabolic disease parameter	Cluster 1 (15)	Cluster 2 (2)	Cluster 3 (15)	Cluster 4 (8)	Cluster 5 (9)
Metritis, %					
Retained placenta, %					
CK, %					
CK and SCK, %					
Hypocalcaemia, %					
Stillbirth, %					
Twins, %					
Dystocia, %					
Metritis 1st lact, %					
Retained placenta 1st lact cows, %					
CK 1st lact cows, %					
CK and SCK 1st lact cows, %					
Hypocalcaemia 1st lact cows, %					
Stillbirth 1st lact cows, %					
Twins 1st lact cows, %					

Dystocia 1st lact cows, %					
Metritis multiparous cows, %					
Retained placenta multiparous cows, %					
CK multiparous cows, %					
CK and SCK multiparous cows, %					
Hypocalcaemia multiparous cows, %					
Stillbirth multiparous cows, %					
Twins multiparous cows, %					
Dystocia multiparous cows, %					
Inc uterine Inv > 30DIM, %					
Inc uterune Inv > 30DIM 1st lact cows, %					
Inc uterine Inv > 30DIM multiparous cows, %					
Pyometra, %					
Pyometra 1st lact cows, %					
Pyometra multiparous cows, %					
Perineal injury, %					
Perineal injury 1st lact cows, %					
Perineal injury multiparous cows, %					
Abomasal pathology, %					

Abomasal pathology 1st lact cows, %					
Abomasal pathology multiparous cows, %					

CK, Clinical ketosis; DIM, Days in milk; lact, lactation; Inc uterine Inv, Incorrect uterine involution; SCK, Subclinical ketosis.

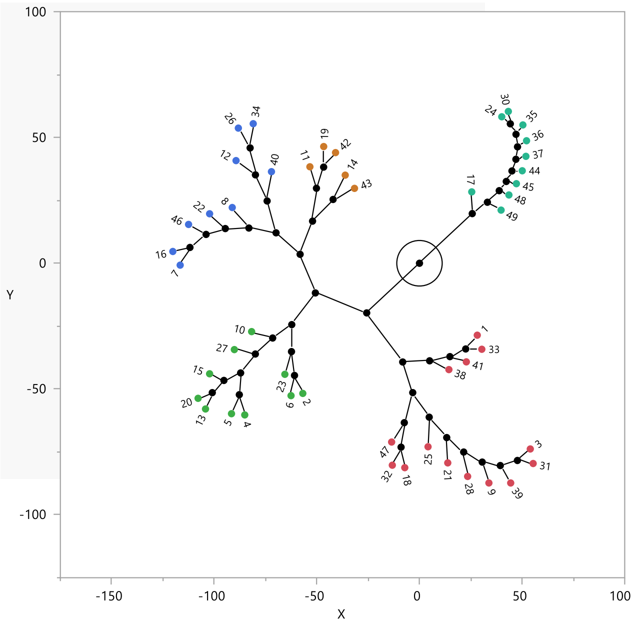

Supplementary Figure 4. Constellation plot showing the results of a hierarchical analysis of the 49 surveys answered of Section 5. Heifers' reproduction. The numeration corresponds to each consultant that answered the survey. Cluster 1, 2, 3, 4 and 5 are represented in red, dark green, blue, brown and soft green color, respectively.

Supplementary Table 5. Answer pattern for the 5 clusters obtained from the 50 parameters evaluated in the Section 5. Heifers' reproduction after hierarchical clustering analysis. It is detailed in brackets the number of surveys answered belonging to each cluster. Parameters considered as highly important, moderately important, low important and irrelevant are showed in green, blue, orange and red, respectively.

Heifers' Reproduction Parameter	Cluster 1 (14)	Cluster 2 (10)	Cluster 3 (9)	Cluster 4 (5)	Cluster 5 (10)
CR, %	green	green	green	green	orange
First service CR, %	green	green	blue	green	red
Interval heat to heat, d	blue	green	blue	green	red
Interval service to service, d	blue	green	blue	green	red
Heat detection rate, %	green	green	green	green	red
Ovarian cysts, %	blue	red	orange	blue	red
Anovulatory heifers, %	green	green	blue	green	red
21d Pregnancy rate, %	green	green	blue	green	red
Service pregnant heifer, n	green	green	green	green	red
Culling rate heifers, %	green	green	blue	blue	red
Heifers culled for reproductive reason, %	green	green	green	green	red
"do not breed" heifers, %	green	green	orange	blue	red
Age at first service, d	green	green	blue	green	red
Age at first calving, d	green	green	green	green	orange
Heifers calving <24 months old, n	green	green	green	orange	red

Heifers calving <23 months old, n					
Heifers calving <22 months old, n					
Heifers calving <21 months old, n					
CR synchronized heifers, %					
CR of the sire, %					
CR of inseminators, %					
Days at pregnancy diagnosis, d					
Number of heifers, n					
Heifers/Cows, %					
Heifers <14 months old, %					
Heifers >14 months old, %					
Heifers <13 months old, %					
Heifers >13 months old, %					
Heifers <12 months old, %					
Heifers >12 months old, %					
Heifers <11 months old, %					
Heifers >11 months old, %					
Heifers >14 months old not serviced, %					
Heifers >13 months old not serviced, %					

Heifers >12 months old not serviced, %					
Heifers >11 months old not serviced, %					
Heifers pregnant, %					
Pregnancy loss, %					
Early pregnancy loss (1-42 days), %					
Pregnancy loss (1-90days), %					
Abortion >90 days, %					
Open heifers > 12 months, %					
Open heifers > 13 months, %					
Open heifers > 14 months, %					
Open heifers > 15 months, %					
Open heifers > 16 months, %					
Open heifers > 17 months, %					
Heifers <2 SD from 400 kg at 400 d, %					
Heifers < 580 kg at calving, %					
Heifer efficiency, %					

CR, Conception rate; SD: Standard deviation.
